# Necroptosis contributes to chronic inflammation and fibrosis in aging liver

**DOI:** 10.1111/acel.13512

**Published:** 2021-11-11

**Authors:** Sabira Mohammed, Nidheesh Thadathil, Ramasamy Selvarani, Evan H. Nicklas, Dawei Wang, Benjamin F. Miller, Arlan Richardson, Sathyaseelan S. Deepa

**Affiliations:** ^1^ Stephenson Cancer Center Oklahoma City OK USA; ^2^ Department of Biochemistry and Molecular Biology Oklahoma City OK USA; ^3^ Oklahoma Center for Geroscience & Brain Aging University of Oklahoma Health Sciences Center Oklahoma City OK USA; ^4^ Aging and Metabolism Research Program Oklahoma Medical Research Foundation Oklahoma City OK USA; ^5^ Oklahoma City VA medical Center Oklahoma City OK USA

**Keywords:** aging, fibrosis, inflammation, liver, necroptosis, necrostatin‐1s

## Abstract

Inflammaging, characterized by an increase in low‐grade chronic inflammation with age, is a hallmark of aging and is strongly associated with various age‐related diseases, including chronic liver disease (CLD) and hepatocellular carcinoma (HCC). Because necroptosis is a cell death pathway that induces inflammation through the release of DAMPs, we tested the hypothesis that age‐associated increase in necroptosis contributes to chronic inflammation in aging liver. Phosphorylation of MLKL and MLKL oligomers, markers of necroptosis, as well as phosphorylation of RIPK3 and RIPK1 were significantly upregulated in the livers of old mice relative to young mice and this increase occurred in the later half of life (i.e., after 18 months of age). Markers of M1 macrophages, expression of pro‐inflammatory cytokines (TNFα, IL6 and IL1β), and markers of fibrosis were all significantly upregulated in the liver with age and the change in necroptosis paralleled the changes in inflammation and fibrosis. Hepatocytes and liver macrophages isolated from old mice showed elevated levels of necroptosis markers as well as increased expression of pro‐inflammatory cytokines relative to young mice. Short‐term treatment with the necroptosis inhibitor, necrostatin‐1s (Nec‐1s), reduced necroptosis, markers of M1 macrophages, fibrosis, and cell senescence as well as reducing the expression of pro‐inflammatory cytokines in the livers of old mice. Thus, our data show for the first time that liver aging is associated with increased necroptosis and necroptosis contributes to chronic inflammation in the liver, which in turn appears to contribute to liver fibrosis and possibly CLD.

## Graphical abstract

Necroptosis increases with age in the liver and paralleled an increase in the expression of proinflammatory cytokines and markers of fibrosis. Hepatocytes and liver macrophages are the major cell types that undergo necroptosis in the livers of old mice. Short term treatment with the necroptosis inhibitor, necrostatin‐1s (Nec‐1s), reduced necroptosis, expression of proinflammatory cytokines and fibrosis in the livers of old mice.

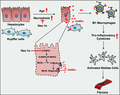

## 1 INTRODUCTION

Aging is characterized by an increase in low‐grade chronic inflammation, termed inflammaging, that is strongly associated with various age‐associated diseases such as type 2 diabetes, cardiovascular disease, cancer, and neurodegenerative diseases such as Alzheimer’s disease. Therefore, inflammaging is considered an important factor in the etiology of these age‐associated diseases (Franceschi & Campisi, 2014). In humans, inflammaging is characterized by an increase in the levels of circulating pro‐inflammatory cytokines interleukin 6 (IL6), tumor necrosis factor‐α (TNFα), and IL‐1β, and increased levels of these cytokines are associated with diseases and mortality (Ferrucci & Fabbri, 2018). Inflammaging is the net effect of multifactorial and multiorgan involvement where immune cells, especially macrophages, in various tissues such as adipose tissue, liver and kidney contribute to the production of pro‐inflammatory cytokines. Pro‐inflammatory cytokines produced by tissues can affect the function of the tissue as well as other distal tissues. For example, pro‐inflammatory cytokine production by adipose tissue increases with age and inhibits the ability of adipose tissue to store fat leading to lipotoxicity in liver and skeletal muscle resulting in hepatic steatosis and muscle dysfunction, respectively (Mancuso & Bouchard, 2019).

Aging is associated with an increase in the levels of inflammatory cytokines in the liver (Stahl et al., 2020). Damage‐associated molecular patterns (DAMPs, example, HMGB1, mitochondrial DNA, nuclear DNA, ATP etc.) released from damaged or dying hepatocytes are one of the proposed mediators of chronic inflammation in the liver (Brenner et al., 2013). Necroptosis, a form of programmed necrosis, is a regulated cell death pathway that is strongly associated with increased inflammation through the release of DAMPs from the necroptotic cells (Newton & Manning, 2016). In contrast, cell death by apoptosis is associated with limited release of DAMPs and therefore is less inflammatory in nature. Absence of caspase‐8, an initiator of extrinsic apoptosis, switches cell death from apoptosis to necroptosis (Fritsch et al., 2019). Necroptosis most likely evolved as an alternative form of cell death to kill cells infected by viral pathogens and to promote inflammatory and immune responses to limit the spread of the viruses (Dondelinger et al., 2016). However, necroptosis is also initiated by necroptotic stimuli [e.g., TNFα, oxidative stress or mTOR/Akt pathway (Royce et al., 2019), which sequentially phosphorylate and activate receptor‐interacting protein kinase 1(RIPK1) and RIPK3 that in turn phosphorylate the pseudokinase mixed lineage kinase domain‐like (MLKL) protein.

Phosphorylation of MLKL leads to its oligomerization, which then binds to and disrupts the cell membrane releasing cellular components including the DAMPs that initiate and exacerbate the inflammatory process. DAMPs released from necroptotic cells bind to pattern recognition receptors (PRRs) such as toll‐like receptors (TLRs) on innate immune cells resulting in the induction of pro‐inflammatory cytokines. Necroptosis has emerged as a novel mode of cell death in various chronic liver diseases such as nonalcoholic fatty liver disease (NAFLD) and nonalcoholic steatohepatitis (NASH), conditions that are also associated with aging in liver (Saeed & Jun, 2014). Various liver cell types such as hepatocytes (Zhong et al., 2020), Kupffer cells (Blériot et al., 2015) and endothelial cells (Zelic et al., 2018) are shown to undergo necroptosis under pathological conditions or injury.

Blocking necroptosis has been shown to reduce hepatic inflammation in mouse models of liver diseases (Majdi et al., 2020; Wu et al., 2020), whereas the role of necroptosis in age‐associated hepatic inflammation is unexplored. Previously, we reported that necroptosis increases with age in the white adipose tissue of mice and is reduced by interventions that delay aging (dietary restriction or in Ames dwarf mice) (Deepa et al., 2018; Royce et al., 2019). Recently, we found that necroptosis and inflammation are increased in the livers of a mouse model of accelerated aging (mice deficient in the antioxidant enzyme, Cu/Zn superoxide dismutase, *Sod1^‐/‐^* mice) and blocking necroptosis reduced inflammation in the livers of *Sod1^‐/‐^* mice (Mohammed et al., 2021). Based on these findings, we hypothesized that age‐associated increase in necroptosis might contribute to the age‐related increase in hepatic inflammation. Because chronic inflammation can lead to liver fibrosis, we also studied the effect of necroptosis on the markers of fibrosis in the liver with age. Our data show that markers of necroptosis, inflammation, and fibrosis increase with age in the livers of mice and blocking necroptosis (using a pharmacological inhibitor of necroptosis, necrostatin‐1s, Nec‐1s) reduced the expression of inflammatory cytokines and fibrosis markers in the livers of old mice.

## 2 RESULTS

### 2.1 Necroptosis markers increase with age in the livers of mice

**FIGURE 1:**
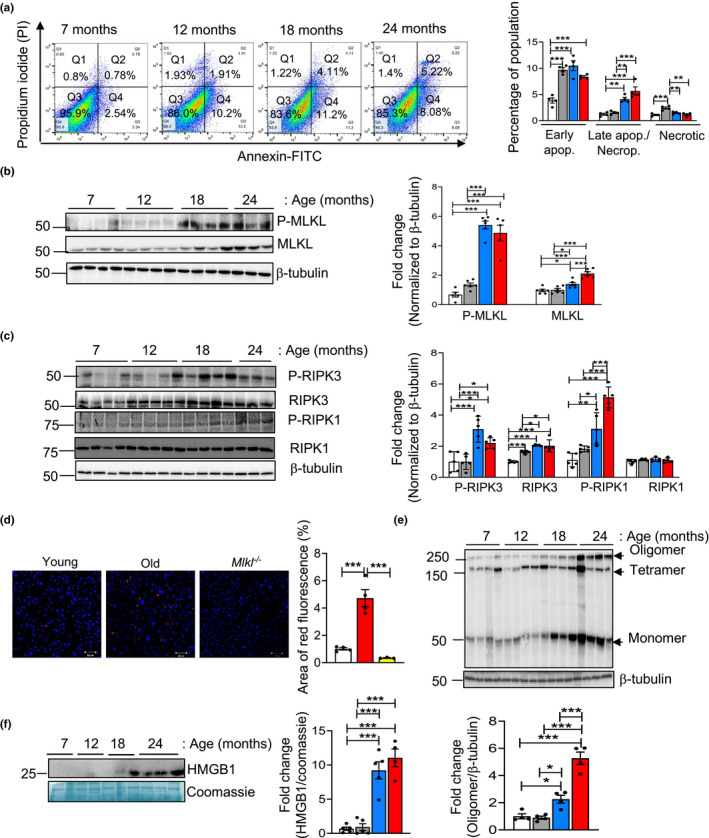
Necroptosis markers increase with age in the livers of mice. (a) Left: Annexin VFITC/PI staining of liver cells from 7, 12, 18, and 22 to 24‐month‐old mice. The upper right quadrant (FITC^+^/PI^+^) represents late apoptotic/necroptotic population, the lower right quadrant (FITC^+^/PI^−^) represents early apoptotic population and the upper left quadrant (FITC^−^/PI^+^) represents the necrotic population. Right: Graphical representation of the early apoptotic, late apoptotic/necroptotic and necrotic populations. (b) Left: Immunoblots of liver extracts prepared from 7 (white bars), 12 (gray bars), 18 (blue bars), and 22 to 24‐month‐old (red bars) mice for PMLKL, MLKL and β‐tubulin. Right: Graphical representation of quantified blots normalized to β‐tubulin. (c) Left: Immunoblots of liver extracts for P‐RIPK3, RIPK3, P‐RIPK1, RIPK1 and β‐tubulin. Right: Graphical representation of quantified blots normalized to β‐tubulin. (d) Left: Immunostaining of liver sections from young (white bar), old (red bar) and *Mlkl* knockout (yellow bar) mice for P‐MLKL. Scale bar: 50 µM. Right: Graphical representation of the percentage of red fluorescent area. (e) Top: Immunoblots probed using anti‐MLKL antibody for oligomers (>250 kDa). β‐tubulin was used as loading control. Bottom: Graphical representation of quantified oligomer normalized to β‐tubulin. (f) Left: Immunoblots of plasma samples for HMGB1. Coomassie stained gel is used as loading control. Right: Graphical representation of quantified blot normalized to Coomassie stained gel. Data represented as mean ± SEM, *p < 0.05, **p < 0.005, ***p < 0.0005, *n* = 5–7 per group

Necroptosis in the livers of mice ranging in age from 7 to 24 months were measured by flow cytometry using Annexin V/propidium iodide (PI) staining and biochemical analysis by western blotting to identify changes in the expression of proteins involved in necroptosis. As shown in Figure 1a, the percent of liver cells undergoing apoptosis (Annexin V positive) and late apoptosis/necroptosis (double positive for Annexin V and PI) were significantly increased with age. The early apoptotic cell population was significantly higher in the liver starting from 12 months of age (2.5‐fold) and remained elevated at 18 (2.7‐fold) and 24 months of age (2.2‐fold), whereas late apoptotic/necroptotic population was significantly higher at 18 and 24 months relative to 7‐month‐old mice (3.3‐ and 4.6‐fold). There was no significant increase in the necrotic population at 18‐ and 24‐month relative to 7‐month‐old mice, however, necrosis was increased at 12 months relative to 7‐month‐old mice. The percent of cells undergoing necroptosis/late apoptosis was twofold higher than the percent of cells undergoing early apoptosis in 24‐month‐old mice.

As flow cytometry cannot differentiate between necroptotic and late apoptotic cell populations, we measured the expression of the three proteins involved in necroptosis. The levels of MLKL and RIPK3 were significantly increased in 18‐and 24‐month‐old mice compared to the 7‐ and 12‐month old mice. However, we observed no changes in the level of RIPK1 with age (Figure 1b, c). The changes in the levels of MLKL and RIPK3 were associated with increased transcript levels of these proteins (Figure S1a). Because the phosphorylation of MLKL, RIPK3 and RIPK1 trigger necroptosis, the levels of phospho‐MLKL (P‐MLKL), P‐RIPK3, and P‐RIPK1 were measured, which showed a significant increase in the 18‐ and 24‐month‐old mice compared to the 7‐ and 12‐month‐old mice (Figure 1b, c). The levels of P‐MLKL and P‐RIPK3 showed a slight reduction at 24 months relative to 18 months, however, this was not statistically significant. In contrast, P‐RIPK1 showed a significant increase at 24 months relative to 18 months. The increased expression of P‐MLKL in the livers of old mice was further confirmed by immunofluorescence staining of young and old mice livers. The specificity of the P‐MLKL staining was confirmed by using liver tissues from old *Mlkl^‐/‐^* mice (Figure 1d). RIPK3‐mediated phosphorylation of MLKL leads to the formation of MLKL tetramers or octamers. For mouse MLKL, tetramers fail to translocate to the plasma membrane and octamer formation is required for pore formation in the membrane (Huang et al., 2017). Consistent with the age‐associated increase in the levels of MLKL, MLKL oligomerization also showed a significant increase with age; levels of MLKL oligomers were similar in the livers of 7‐ and 12‐month‐old mice, whereas MLKL oligomer levels were significantly increased by twofold in 18‐ and fivefold in 24‐month‐old mice, compared to 7‐ or 12‐month‐old mice (Figure 1e).

Because DAMPs are released from cells undergoing necroptosis, we measured the levels of circulating levels of high‐mobility group box‐1 (HMGB1) protein, a DAMP that has been shown to be released by hepatic necroptosis and is associated with acute liver injury and chronic liver disease (Wen et al., 2020). Circulating levels of HMGB1 are significantly elevated at 18 months (fourfold) and 24 months (sevenfold) of age relative to 7‐ or 12‐month‐old mice (Figure 1f). The changes in circulating HMGB1 levels parallel the changes in the necroptosis markers in the liver, providing additional data supporting that necroptosis increases with age in liver.

**FIGURE 2:**
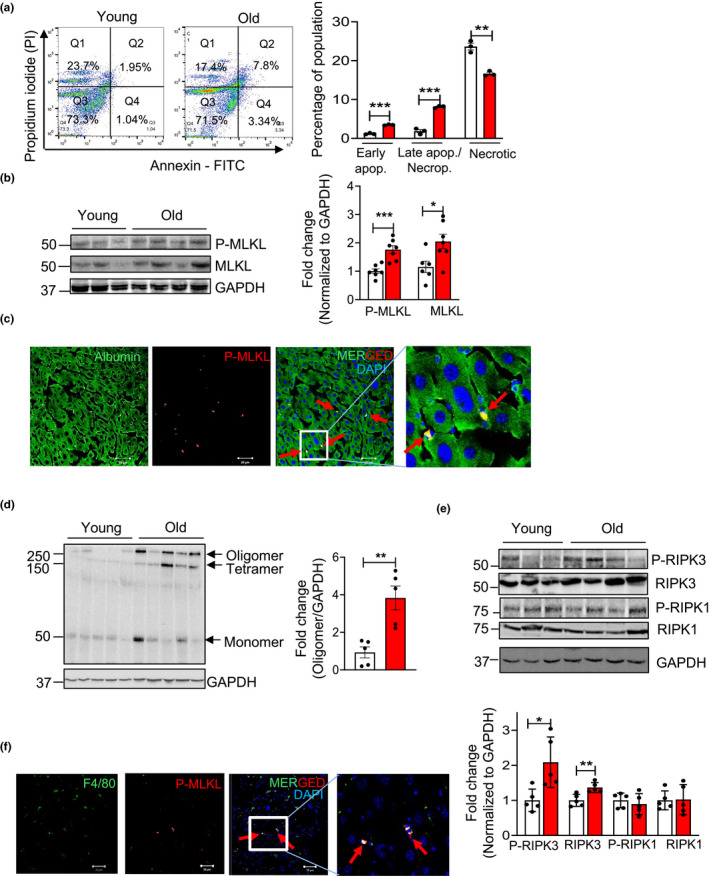
Increased expression of necroptosis markers in hepatocytes isolated from old mice. (a) Left: Representative image of Annexin V/PI staining of hepatocytes isolated from young (white bars) and old mice (red bars). The upper right quadrant (FITC^+^/PI^+^) represents late apoptotic/necroptotic population, the lower right quadrant (FITC^+^/PI^−^) represents early apoptotic population and the upper left quadrant represents necrotic population. Right: Graphical representation of the early apoptotic, late apoptotic/necroptotic and necrotic populations. (b) Left: Immunoblots of hepatocytes isolated from young and old mice for P‐MLKL, MLKL and GAPDH. Right: Graphical representation of quantified blot normalized to GAPDH. (c) Immunostaining for the albumin (green) and P‐MLKL (red) in the livers from old mice. Arrows indicate co‐localization (yellow). Scale bar: 50 µM. (d) Left: Immunoblots of isolated hepatocytes from young and old mice for MLKL oligomer. Right: Graphical representation of quantified oligomer band normalized to GAPDH. (e) Top: Immunoblots of isolated hepatocytes from young and old mice for P‐RIPK3, RIPK3, P‐RIPK1, RIPK1 and GAPDH. Bottom: Graphical representation of quantified blots normalized to GAPDH. (f) Immunostaining for F4/80 (green) and P‐MLKL (red) in livers from old mice. Arrows indicate co‐localization (yellow). Scale bar: 50 µM. Data represented as mean ± SEM, *p < 0.05, **p < 0.005, ***p < 0.0005, *n* = 5–7/group

Hepatocytes constitute 80% of the liver mass and are reported to undergo cell death in CLD (Shojaie et al., 2020); therefore, we tested whether hepatocytes were undergoing increased necroptosis. Hepatocytes were isolated from young (7‐month) and old (24‐month) mice and purity of the isolated hepatocytes were confirmed by western blotting as shown in Figure S1b and Figure S1c in the supplement. Flow cytometry data of isolated hepatocytes showed significant increase in apoptotic (threefold) and necroptosis/late apoptosis (4.4‐fold) populations from old mice relative to young mice, whereas the percentage of necrotic cell population was significantly reduced in hepatocytes from old mice (Figure 2a). Consistent with this, levels of P‐MLKL and MLKL were both significantly elevated (twofold) in the hepatocytes isolated from old mice relative to young mice (Figure 2b). This was further confirmed by co‐immunostaining of liver sections from old mice with P‐MLKL and albumin, which showed that P‐MLKL colocalizes with albumin (Figure 2c). Similarly, levels of MLKL oligomers were significantly elevated (fourfold) in hepatocytes isolated from old mice (Figure 2d). Levels of P‐RIPK3 and RIPK3 are significantly elevated in the hepatocytes from old mice (twofold and 1.4‐fold respectively), whereas levels of P‐RIPK1 and RIPK1 were similar in hepatocytes from young and old mice (Figure 2e). *Mlkl* and *Ripk3* transcript levels are also significantly elevated (7.5‐fold and eightfold), whereas *Ripk1* transcript levels were similar, in the hepatocytes of old mice relative to young mice (Figure S1d).

Because Kupffer cells are reported to undergo necroptosis (Blériot et al., 2015), necroptosis in liver macrophages in aging was assessed. Double immunostaining using P‐ MLKL and F4/80 (macrophage marker) showed co‐localization of these two proteins in the livers of old mice (Figure 2f). The macrophage population isolated from old mice livers also showed a significant increase in *Mlkl* transcripts (twofold) relative to young, similar to hepatocytes (Figure S1e). Flow cytometry data also showed a significant increase (1.7‐fold) in the late apoptotic/necroptotic population in the Kupffer cell fraction isolated from the livers of old mice (Figure S1f and Figure S1g).

### 2.2 Age‐associated increase in chronic inflammation in the livers of mice

**FIGURE 3:**
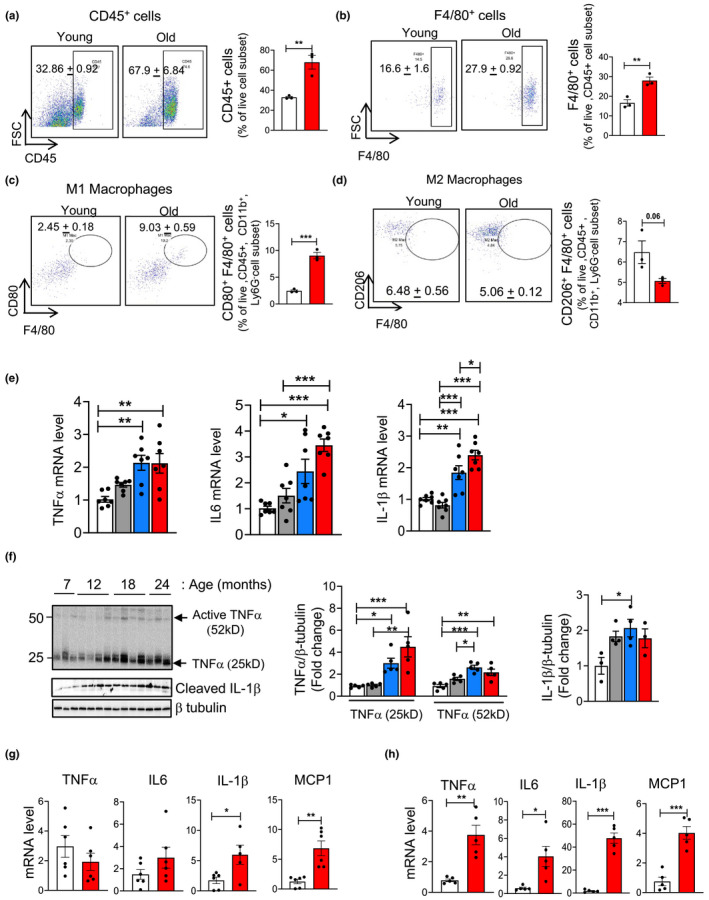
Markers of inflammation increase with age in the livers of mice. (a) Left: Representative flow cytometric analysis of CD45^+^ cells. Right: Graphical representation of the percentage population of CD45^+^ cells in the liver of young (white bar) and old mice (red bar), gated on live cell population. (b) Left: Representative flow cytometric analysis of F4/80^+^ cells. Right: Graphical representation of the percentage population of F4/80^+^ cells in the livers of young and old mice, gated on live, CD45^+^ cells. (c) Left: Representative flow cytometric analysis of M1 macrophages (CD80^+^ F4/80^+^ cells). Right: Graphical representation of the percentage population of M1 macrophages (CD80^+^ F4/80^+^ cells) in the livers of young and old mice, gated on live, CD45^+^ CD11b^+^ Ly6G^−^ cells. (d) Left: Representative flow cytometric analysis of M2 macrophages (CD206^+^ F4/80^+^ cells). Right panel: Graphical representation of the percentage population of M1 macrophages (CD206^+^ F4/80^+^ cells) in the livers of young and old mice, gated on live, CD45^+^ CD11b^+^ Ly6G^−^ cells. (e) Transcript levels of TNFα, IL6, IL‐1β in the livers of 7 (white bars), 12 (gray bars), 18 (blue bars), and 22 to 24‐month‐old (red bars) mice normalized to β‐ microglobulin and expressed as fold change. (f) Left: Immunoblots of liver extracts for TNFα, cleaved IL‐1β and β‐tubulin. Right: Graphical representation of quantified blots normalized to β‐tubulin. Transcript levels of TNFα, IL6, IL‐1β and MCP‐1 in isolated F4/80+ cells (g) hepatocytes (h) from young and old mice normalized to β‐microglobulin and expressed as fold change. Data represented as mean ± SEM, *p < 0.05, **p < 0.005, ***p < 0.0005, *n* = 5–7/group

Damage‐associated molecular patterns released from cells undergoing necroptosis bind to cell surface receptors on innate immune cells (e.g., macrophages) to induce an inflammatory response through the production of pro‐inflammatory cytokines (Newton & Manning, 2016). Therefore, we measured changes in liver macrophages with age as well as levels of pro‐inflammatory cytokines in the liver, isolated hepatocytes and macrophage populations. Flow cytometry analysis showed that the total immune cell population (percentage of CD45^+^ cells), was significantly increased in the livers of old mice (67.9% ± 6.84%) relative to young mice (32.86% ± 0.92%) (Figure 3a). Next, the total macrophage population was analyzed by staining the cells with F4/80, a cell surface marker expressed by liver macrophages. A significant increase in the F4/80^+^ cell population was observed in the livers of old mice (27.9% ± 1.8%) relative to young mice (16.6% ± 1.6%) (Figure 3b). Transcript levels of F4/80 and monocyte chemoattractant protein‐1 (MCP‐1) were similar in 7‐ and 12‐month‐old mice, whereas the levels are significantly increased (twofold for F4/80 and fivefold and sixfold for MCP‐1) in 18‐ and 24‐month‐old mice. (Figure S2a). These data are consistent with macrophage number in the liver increasing with age.

Macrophages are categorized into M1 or M2 phenotypes: M1 macrophages play a more pro‐inflammatory role in liver injury and M2 macrophages exert an anti‐inflammatory effect. Flow cytometric analysis of liver cells showed that M1 macrophage population (CD45^+^CD11b^+^Ly6G^‐^CD80^+^F4/80^+^ cells was significantly increased in the livers of old mice (9.03% ± 0.59%) relative to young mice (2.45% ± 0.18%) (Figure 3c). The M2 macrophage population (CD45^+^CD11b^+^Ly6G^‐^ F4/80^+^ CD206^+^ cells) was reduced in the livers of old mice (5.06 ± 0.12) relative to young mice (6.48 ± 0.56), however, this reduction did not reach statistical significance (Figure 3d). The transcript levels of M1 macrophage markers CD68, CD86, TLR4, and CD11c were significantly upregulated at 18 months compared to their expression at 7 or 12‐months of age. Expression of M1 macrophage markers at 24 months was similar to those at 18‐months, except for CD11c, which was increased between 18 and 24 months of age (Figure S2b). The transcript levels of M2 macrophage markers (Arg1 and Fizz1) are significantly reduced at 12,18, and 24 months compared to their expression at 7 months of age (Figure S2c).

Consistent with the increase in M1 macrophage markers, expression of pro‐inflammatory cytokines TNFα, IL6 and IL‐1β were significantly upregulated (twofold to threefold) in the livers at 18‐ and 24‐month relative to 7‐ or 12‐month (Figure 3e). The changes in protein levels of TNFα and IL‐1β also were significantly increased in the livers of 18‐ and 24‐month‐old mice relative to 7‐ or 12‐month old mice (Figure 3f). Analysis of the isolated macrophage population (F4/80^+^ fraction) showed a significant increase in the transcript levels of IL‐1β (3.5‐fold) and MCP‐1 (5.6‐fold) in old mice relative to young mice (Figure 3g). However, transcript levels of TNFα or IL6 in the F4/80^+^ fraction of cells were similar in young and old mice.

Because hepatocytes are also reported to produce pro‐inflammatory cytokines (Stahl et al., 2020), we measured the expression of pro‐inflammatory cytokines in isolated hepatocytes. Transcript levels were significantly increased for TNFα (fourfold), IL6 (fourfold) and IL‐1β (40‐fold) and MCP1/CCL2 (fourfold) in the hepatocytes isolated from old mice relative to hepatocytes from young mice (Figure 3h). Liver sinusoidal endothelial cells (LSEC) are known to produce inflammatory cytokines in chronic liver diseases (Wang & Peng, 2021), however, TNFα, IL6, IL1β, and MCP1 were similar in isolated LSEC from young and old mice livers (Figure S2d).

### 2.3 Age‐associated increase in fibrosis in the livers of wild‐type mice

**FIGURE 4:**
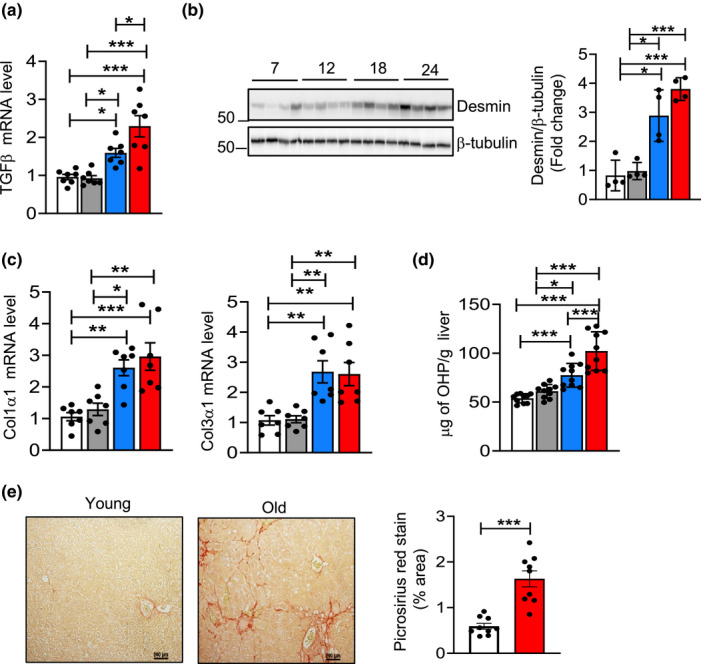
Markers of fibrosis increase with age in the livers of mice. (a) Transcript levels of TGFβ in liver of 7 (white bars), 12 (gray bars), 18 (blue bars), and 22 to 24‐month‐old mice (red bars) normalized to β‐microglobulin and expressed as fold change. (b) Left: Immunoblots of liver extracts for desmin and β‐tubulin. Right: Graphical representation of quantified blots normalized to β‐tubulin. (c) Transcript levels of Col1α1, Col3α1 in livers normalized to β‐microglobulin and expressed as fold change. (d) Levels of hydroxyproline in the livers, expressed as microgram of hydroxyproline/g of liver tissue. (e) Left: Picrosirius red staining in young and old mice. Scale bar: 200µm. Right: Quantification of fibrotic area in young (white bar) and old (red bar) mice. Data represented as mean ± SEM, *p < 0.05, **p < 0.005, ***p < 0.0005, *n* = 7–10/group

Chronic hepatic inflammation is a major driver of liver fibrosis, and the trans differentiation of quiescent hepatic stellate cells (HSCs) drives fibrogenesis. Transforming growth factor beta (TGFβ) is a major player in the induction of fibrosis and is produced by several liver cells such as LSEC, Kupffer cells, platelets, hepatocytes and macrophages (Fabregat and Caballero‐Díaz, 2018). The transcript levels TGFβ expression was significantly increased (1.5‐ and twofold) in the livers of 18‐ and 24‐month‐old mice relative to its expression in the livers of 7‐ or 12‐month‐old mice (Figure 4a). Desmin, a protein produced by activated HSCs, which is strongly upregulated in liver fibrosis was threefold to fourfold higher at 18‐ and 24‐month relative to 7‐ or 12‐month (Figure 4b). Consistent with this, the expression Col1α1 and Col3α1 were significantly increased (2.5fold) in the livers at 18‐ and 24‐month relative to 7 or 12 months (Figure 4c). Next, we assessed total collagen content by measuring concentration of hydroxyproline (OHP) because total collagen content is an indicator of the severity of fibrosis. At 18 and 24 months of age, OHP content in the liver was significantly higher (1.5‐ and 1.9‐fold) relative to 7‐ or 12‐month‐old mice (Figure 4d). Consistent with this, picrosirius red (PSR) staining in the old mice liver was significantly higher (2.7‐fold) relative to young mice, suggesting increased levels of collagen in old mice liver (Figure 4e). Thus, markers of fibrosis show an age‐associated increase in livers of mice that paralleled the changes in inflammation and necroptosis.

### 2.4 Blocking necroptosis reduces hepatic inflammation and fibrosis

**FIGURE 5:**
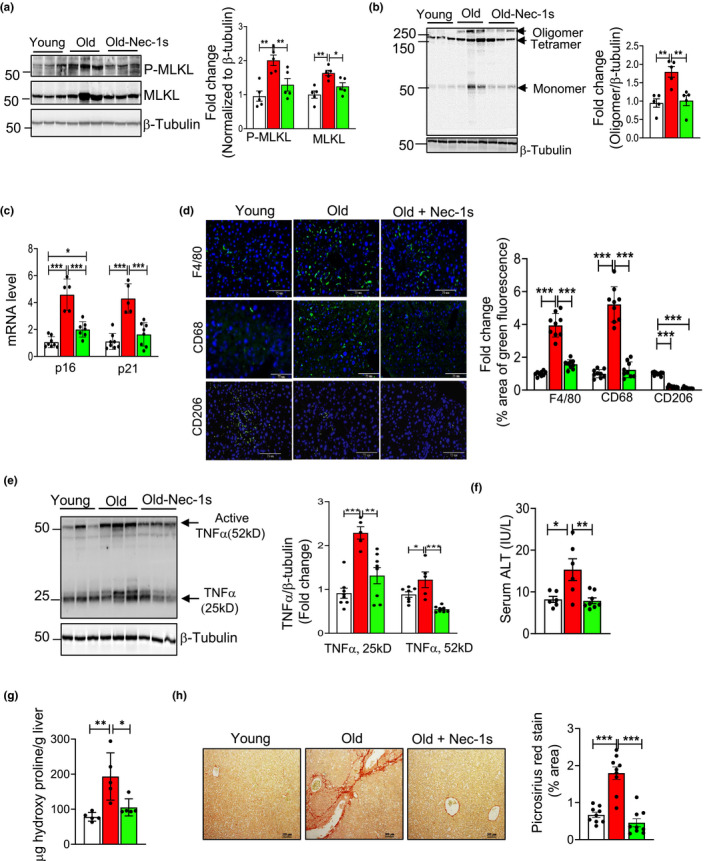
Nec‐1s treatment reduces markers of necroptosis, inflammation, fibrosis, and hepatic damage in the livers of old mice. (a) Left: Immunoblots of liver extracts from young (7‐month, white bars), old (24‐month, red bars), and old mice treated with Nec‐1s (24 months, old‐Nec‐1s, green bars) for P‐MLKL, MLKL and β‐tubulin. Right: Graphical representation of quantified blots normalized to β‐tubulin. (b) Left: Immunoblots of non‐reduced samples for MLKL oligomer and β‐tubulin. Right: Graphical representation of quantified oligomer normalized to β‐tubulin. (c) Transcript levels of p16 and p21 normalized to β‐microglobulin and expressed as fold change. (d) Left: Immunostaining for F4/80, CD68 and CD206 (green). Nucleus counterstained with DAPI (blue). Scale bar: 75 µM. Right: Graphical representation of the percentage area of green fluorescence. (e) Left: Immunoblots of liver extracts for TNFα and β‐tubulin. Right: Graphical representation of quantified blots normalized to β‐tubulin. (f) ALT activity measured in the serum expressed as IU/L. (g) Levels of OHP expressed as microgram of hydroxyproline/g of liver tissue. (h) Left: Picrosirius red staining. Scale bar: 200 µm. Right: Quantification of staining. Data represented as mean ± SEM, *p < 0.05, **p < 0.005, ***p < 0.0005, *n* = 5–7/group

To determine if necroptosis was responsible for the increased inflammation and fibrosis seen in the livers of old mice, we tested the effect of blocking necroptosis on hepatic inflammation and fibrosis in old mice. Mice were treated with Nec‐1s (RIPK1 inhibitor) for 30 days, which has been shown to effectively block necroptosis and reduce inflammation in the liver as well as in other tissues (Mohammed et al., 2021). Nec‐1s treatment had no effect on the body weight or liver weight of old WT mice (Figure S3a and Figure S3b), indicating no obvious negative effect of Nec‐1s on old mice. Nec‐1s treatment significantly reduced necroptosis as predicted, that is, the levels of PMLKL and MLKL oligomers were reduced to the levels seen in young mice (Figure 5a, b). Because apoptosis is increased in livers of old mice and Nec‐1s inhibits RIPK1, which is known to be involved in apoptosis, we checked whether Nec‐1s has an effect on apoptosis by measuring the expression of cleaved Caspase‐3. As seen in Figure S3c, Nec‐1s did not exert an effect on apoptosis. To test whether Nec‐1s had any effect on other pathways, we assessed changes in cell senescence by measuring p16 and p21 expression, which have been reported to increase with age (Franceschi & Campisi, 2014). As shown in Figure 5c, we observed a fourfold to fivefold increase in the transcript levels of p16 and p21 in the livers of the old mice compared to young mice. To our surprise, we found that transcript levels of p16 and p21 were significantly reduced after Nec‐1s treatment to levels similar to that found in young mice.

Next, we measured the effect of Nec‐1s treatment on the age‐related increase in macrophage number, macrophage activation, and hepatic inflammation. Transcript levels of F4/80, MCP1 and M1 macrophage markers that were elevated in the livers of old WT mice were significantly reduced by Nec‐1s to levels present in the young WT mice (Figure S3d and Figure S3e), whereas expression of M2 macrophage markers was unaffected by Nec‐1s treatment (Figure S3f). Consistent with this, protein expression of expression of F4/80 (fourfold) and CD68 (fivefold) was significantly increased in the livers of old mice, which were significantly reduced by Nec‐1s treatment to levels similar to young liver as shown by immunofluorescence staining (Figure 5d). In contrast, the expression of CD206 protein (M2 macrophage marker) was significantly reduced (80%) in the livers of old mice relative to young mice and Nec‐1s had no effect on CD206 expression in the livers of old mice (Figure 5d). Similarly, levels of transcripts for pro‐inflammatory cytokines TNFα, IL6 and IL‐1β, which were elevated in the livers of old WT mice, were significantly reduced by Nec‐1s treatment and were comparable to the levels in young mice (Figure S3g). Consistent with the reduction in transcript levels, protein levels of TNFα in the livers of old WT mice were also significantly reduced by Nec‐1s treatment (Figure 5e).

Finally, we determined the effect of blocking necroptosis on liver damage and fibrosis. Liver damage was assessed by measuring circulating levels of alanine aminotransferase (ALT). We found a significant increase in the serum levels of ALT in the old mice (2.2‐fold) relative to young mice, and Nec‐1s treatment significantly reduced serum ALT levels in old mice to levels comparable to young mice (Figure 5f). Markers of fibrosis, which were higher in the livers of old WT mice, were significantly reduced by Nec‐1s (Figure S3h and Figure 5g). To check for the effect of Nec‐1s on deposition of collagen fibers, Picrosirius red staining was done (Figure 5h). Consistent with the effect of Nec‐1s on the markers of fibrosis, we saw that Nec‐1s reduced the increased collagen deposition in old mice (2.5 fold) to levels similar to that of young mice. Thus, our data shows that short‐term Nec‐1s treatment can exert its effect on the M1 macrophages and reduce age‐associated hepatic inflammation and thereby liver damage and fibrosis.

## 3 DISCUSSION

The goal of this study was to determine if necroptosis plays a role in the age‐related increase in inflammation in liver of mice. We looked at a range of ages to delineate the progression of necroptosis and inflammation. Our data show that all the markers of necroptosis (e.g., P‐MLKL and P‐RIPK3) increased with age in liver. The increase occurred primarily in the later third of life, that is, after 18 months of age. Additional evidence that necroptosis increased with age was our observation of an age‐related increase in MLKL oligomers, which bind and permeabilizes the cell membrane resulting in the release of DAMPs (Newton & Manning, 2016), and the age‐related increase in the DAMP, HMGB1 in the plasma. While the expression of P‐MLKL was similar at 18 and 24 months, MLKL oligomer levels were significantly increased from 18 to 24 months. It is possible that levels/activities of HSP70 or TAM kinases, the regulators of MLKL oligomerization, might be regulating MLKL oligomerization at 24 months (Meng et al., 2021). In addition to necroptosis, RIPK1 is also involved in apoptosis as well as cell survival pathways such as NF‐kB, Akt, and JNK (Mifflin et al., 2020). Therefore, it is possible that an increase in P‐RIPK1 at 24 months might reflect the involvement of P‐RIPK1in the other cellular pathways. We found that markers of necroptosis were increased in hepatocytes and macrophages in liver with age. Previous studies reported that hepatocyte necroptosis is increased by a number of conditions, for example, NAFLD (Wu et al., 2020). Similarly, liver macrophages have also been reported to undergo necroptosis (Blériot et al., 2015). However, this is the first report showing that necroptosis is increased with age in both hepatocytes and macrophages.

Several factors could lead to the age‐related increase in necroptosis. Circulating levels of TNFα, a well‐known inducer of necroptosis (Degterev et al., 2005), have been reported to increase with age in humans (Bruunsgaard et al., 2003). In addition, oxidative stress, which has been shown to increase with age in a wide variety of tissues including liver has been shown to induce necroptosis in both cell cultures and animals (Royce et al., 2019). In addition, *Sod1^‐/‐^* mice, which show a dramatic increase in oxidative stress, have increased expression of necroptosis markers in the liver (Mohammed et al., 2021) and white adipose tissue (Royce et al., 2019). Mammalian target of rapamycin (mTOR) signaling has been associated with increased necroptosis and mTOR signaling has been shown to increase with age in various tissues including liver and to be associated with various age‐related diseases (Royce et al., 2019). Therefore, it is possible that the age‐associated increase in TNFα, oxidative stress and mTOR activation makes cells in old mice more prone to undergo necroptosis.

Although it is well established that chronic inflammation increases with age (inflammaging) and is associated with a large number of age‐related diseases, the pathways/processes responsible for inflammaging are unknown. We wanted to determine if the increased necroptosis we observed in liver resulted in increased inflammation because necroptosis is a cell death pathway that can induce inflammation through the release of DAMPs from the necroptotic cells. We observed a strong age‐related association between necroptosis and inflammation in liver as both increase in the latter half of life, around 18 months of age when mice began to exhibit some age‐associated pathologies (Stahl et al., 2020). For example, the total macrophage content as well as pro‐inflammatory M1 macrophages increase with age, whereas anti‐inflammatory M2 macrophages decline with age. Consistent with the increase in M1 macrophages, expression of key pro‐inflammatory cytokines associated with inflammaging (e.g., TNFα, IL6 and IL‐1β) also showed an increase in the livers of old mice. We also found that pro‐inflammatory cytokine/chemokine expression (e.g., TNFα, IL6, IL1β, and CCL2) were increased in isolated hepatocytes, isolated macrophages (IL1β and CCL2), but not in LSECs from old mice. Thus, both liver macrophages and hepatocytes are potential sources of inflammatory cytokines seen in the livers of old mice. PRRs such as TLRs are expressed in many liver cell types such as Kupffer cells, monocyte derived macrophages, HSC and LSEC (Faure‐Dupuy et al., 2018). Therefore, it is possible that DAMPs released from necroptotic cells could be sensed by different cell types in the liver. Future studies will address the in vitro effects of DAMPs on inflammatory cytokine production by different liver cell types.

The strongest evidence for necroptosis playing a role in inflammaging in the liver is our data with the RIPK1 inhibitor, Nec‐1s. Treating old mice with Nec‐1s for 30 days reduced markers of necroptosis and MLKL oligomerization to levels observed in young mice. Importantly, we observed that Nec‐1s treatment of old mice resulted in reduced M1 macrophages and the expression of pro‐inflammatory cytokines. Interestingly, Nec‐1s treatment had no effect on the levels of anti‐inflammatory M2 macrophages, suggesting that reduction of M1 macrophages could be the possible mechanism for reduced inflammation observed with Nec‐1s treatment. Because DAMPs are one of the factors that are known initiate macrophages activation and polarization (Lee et al., 2020), a reduction in DAMPs release due to necroptosis inhibition might contribute to the reduction in M1 macrophage markers. It is also possible that reduction in CCL2 with Nec‐1s treatment could reduce monocyte infiltration, thereby reducing the M1 macrophage pool. For example, RIPA‐56, a necroptosis inhibitor, has been shown to reduce monocyte infiltration to spinal cord in a mouse model of multiple sclerosis (Zhang et al., 2019). Consistent with our data that necroptosis plays a role in inflammaging in the liver are previous data with *Sod1^‐/‐^* mice, which show accelerated aging. Short‐term Nec‐1s treatment effectively reduced necroptosis and inflammation in the livers of *Sod1^‐/‐^* mice (Mohammed et al., 2021). In addition, Nec‐1s treatment has been reported to reduce inflammation associated with necroptosis in multiple sclerosis and amyotrophic lateral sclerosis (Mifflin et al., 2020).

Nec‐1s is a specific and potent inhibitor of RIPK1 (Takahashi et al., 2012) with no reported off target effects. Because RIPK1 is involved in apoptosis as well as necroptosis, the effect of Nec‐1s on inflammation could also arise from alterations in apoptosis. However, we found that Nec‐1s treatment had no effect on apoptosis in the livers of old mice. Cell senescence has been proposed to play a role in inflammaging (Franceschi & Campisi, 2014), and Krishnamurthy et al. (2004) have shown that cell senescence is increased with age in liver. Therefore, we measured the levels of p16 and p21 transcripts in the livers of old mice treated with Nec‐1s and found the increase in p16 and p21 in old mice was attenuated by Nec‐1s treatment. Thus, the reduced inflammation in the livers of old mice could arise not only from reduced necroptosis but also cell senescence. These surprising data also indicate that necroptosis may play a role in cell senescence. For example, DAMPs produced by necroptotic cells might have a by‐stander effect and push neighboring cells to become senescent. Therefore, the reduction in necroptosis/DAMPs by Nec‐1s treatment could result in a reduced generation of senescent cells. On the other hand, reducing necroptosis could play a role in the removal of senescent cells through its action on macrophages, which play an important role in removal of senescent cells (Kale et al., 2020). Exposure to chronic inflammation has been shown to alter macrophage function leading to a reduction in their ability to remove cells, such as bacteria and senescent cells, and increase their production of inflammatory cytokines (Thevaranjan et al., 2017). Because Nec‐1s treatment reduced inflammation and M1 macrophages, it is possible that the reduction in cell senescence by Nec‐1s treatment arose from increased clearance of senescent cells. Although Nec‐1s is specific inhibitor of RIPK1, we cannot rule out off target effects. Therefore, future studies will include other pharmacological compounds that inhibit necroptosis or using genetic knockout models of *Ripk3* or *Mlkl*.

We were also interested in determining if necroptosis and chronic inflammation had any functional/pathological importance in liver. CLD, especially fibrosis has been reported to increase with age in the livers of mice and humans (Kim et al., 2015) and is associated with increased inflammation. Therefore, we measured various markers of CLD in the livers of the old mice and found that inhibiting necroptosis and reducing inflammation in the livers of old mice reduced markers of CLD such as serum ALT levels and fibrosis. These data are consistent with previous reports showing that blocking necroptosis genetically (*Ripk3^‐/‐^*) in mice fed a methionine‐deficient diet or pharmacologically (RIPA56) in mice fed a high‐fat diet reduced liver fibrosis (Gautheron et al., 2014; Majdi et al., 2020).

In summary, our data proved strong support for necroptosis playing a role in inflammaging in the liver and the age‐related increase in CLD. Our data are also translationally important because it suggests that pharmacologically inhibiting necroptosis could be an effective strategy to reduce inflammaging and age‐related diseases in which chronic inflammation plays a role, for example, cancer, Alzheimer’s disease, cardiovascular disease, etc. Currently, therapeutic applications of RIPK1 inhibitors for the treatment of a variety of human diseases are being tested in clinical trials (Mifflin et al., 2020). In addition, several FDA‐approved anti‐cancer drugs such as Sorafenib and Dabrafenib have been identified as anti‐necroptotic agents (Fulda, 2018). While the adverse effect of necroptosis inhibition is not clear, it is highly unlikely that inhibiting necroptosis by targeting RIPK1 will increase the susceptibility to viral infection because virus‐induced necroptosis occurs through the activation of RIPK3‐MLKL pathway, which is independent of RIPK1. Similarly, it will be difficult to predict whether necroptosis inhibition will have a pro‐tumorigenic effect. Necroptosis has been reported to exhibit dual effects in cancer, that is, necroptosis can either promote or reduce tumor growth depending on the type of cancer (Gong et al., 2019). Because aging is a slow and gradual process, further studies with long‐term Nec‐1s treatment will be needed to address the feasibility of using necroptosis inhibitor(s) for inflammaging and aging. Currently, we are conducting the first study to determine if blocking necroptosis genetically over the lifespan of a mouse will significantly improve the healthspan, reduce age‐related pathologies and increase lifespan.

In conclusion, our study shows that necroptosis is a potential contributor to age‐associated chronic inflammation and fibrosis in the liver. Thus, necroptosis is a potential therapeutic target for treating chronic liver diseases associated with aging.

## 4 EXPERIMENTAL PROCEDURES

### 4.1 Animals

All procedures were approved by the Institutional Animal Care and Use Committee at the University of Oklahoma Health Sciences Center (OUHSC). C57BL/6 male wild‐type (WT) mice of different age groups (7‐, 12‐, 18‐, and 22 to 24‐month‐old) (*n* = 5–10/group) were received from National Institute on Aging. After receiving the mice, they were group housed as received in ventilated cages 20  ±  2°C, 12‐h/12‐h dark/light cycle and were fed rodent chow (5053 Pico Lab, Purina Mills, Richmond, IN) *ad libitum* at the OUHSC animal care facility for 2 weeks before euthanizing the mice for tissue collection.

### 4.2 Annexin V/Propidium iodide (PI) staining of whole liver tissue

Mice were euthanized and in vivo perfusion was performed as described by (Liu et al., 2017) and annexin PI staining was performed as described in Mohammed et al. (2019) and analyzed using FACS Calibur flow cytometer (BD Biosciences). The data were analyzed using Flow Jo (BD Biosciences) software.

### 4.3 Isolation of hepatocytes

Hepatocyte isolation was performed as described by Liu et al. (2017). The purity of the isolated hepatocytes was checked by real‐time PCR and western blotting for the expression of liver cell specific markers: albumin (hepatocytes), F4/80 (macrophages), Clec4f (Kupffer cells), CD31 (endothelial cells) and desmin (hepatic stellate cells).

### 4.4 Quantitative real‐time PCR (RT‐PCR)

Real‐time‐PCR was performed using 20 mg frozen liver tissues as described previously (Mohammed et al., 2021). The list of primers used are given in Table S1.

### 4.5 Western Blotting

Western blotting was performed as described previously (Mohammed et al., 2021). Images were taken using a Chemidoc imager (Bio‐Rad) and quantified using ImageJ software (U.S. National Institutes of Health). Primary antibodies against the following proteins were used: phospho(S345) MLKL, phospho(T231+ S232)‐RIPK3, HMGB1 and TNFα from Abcam; RIPK1 and RIPK3 from Novus Biologicals; MLKL from Millipore Sigma; Phospho (Ser166)‐RIPK1, Cleaved Caspase‐3, Caspase 3 and Cleaved IL‐1β from Cell Signaling Technology (Danvers, MA); desmin from ThermoFisher Scientific; GAPDH, β‐tubulin and β‐actin were from Sigma‐Aldrich. HRP‐linked anti‐rabbit IgG, HRP‐linked anti‐mouse IgG and HRP‐linked anti‐rat IgG from Cell Signaling Technology (Danvers, MA) were used as secondary antibodies.

### 4.6 Detection of MLKL oligomers by western blotting

Mixed lineage kinase domain‐like oligomerization in liver was detected by western blotting under non‐reducing conditions (Wu et al., 2020).

### 4.7 Western blotting for HMGB1

Plasma concentration of HMGB1 was analyzed by western blotting as described by Higgins et al., (2013).

### 4.8 Magnetic activated cell sorting (MACS) for liver macrophages and LSEC

The different hepatic cell populations were isolated by MACS as described by Liu et al. (2017). After isolation of hepatocyte fraction, magnetically labeled CD146 antibody (Miltenyi) was added to non‐parenchymal fraction and LSEC fraction was collected by passing through a magnetic column as per manufacturer’s instructions. The CD146^‐^ fraction was incubated with biotin labeled F4/80 antibody (Miltenyi) and anti‐biotin microbeads, and magnetic separation was performed to isolate the F4/80^+^ cell fraction. The purity of the isolated fractions was checked by real‐time PCR and western blotting for cell specific markers.

### 4.9 Characterization of M1/M2 macrophages by flow cytometry

Immune cell population was analyzed as described by Mohar et al., (2015) with some modifications. Hepatocytes and non‐parenchymal cells (NPC) were separated as described above. The pelleted NPC was purified by density gradient centrifugation using OptiPrep density gradient media (Cosmo Bio USA) and interphase was used for flow cytometric analysis. Live cell gating was done with Live/Dead fixable violet dead cell stain kit from Thermo Fisher Scientific. The following antibodies were used for staining (Biolegend, SanDiego, CA): CD45‐ APC/Cy7, CD11b‐PE/Cy7, Ly6G‐FITC, F4/80‐PE, CD80‐PE/Cy5, CD206PE Dazzle, CD16/32. Data was collected using Stratedigm 4‐Laser flowcytometer and analyzed using Flow Jo software (BD Biosciences) software. The gating strategy that was followed for the analysis is included in Figure S4.

### 4.10 Hydroxy proline (OHP) assay

Hydroxy proline was performed as described by Smith et al., (2016). The OD values were converted into μg units using the 4‐parameter standard curve generated using the standards and expressed as μg/g of tissue.

### 4.11 Picrosirius red staining

Picrosirius red staining was done using standardized protocol at the Imaging Core facility at the Oklahoma Medical Research Foundation. The images were taken using a Nikon Ti Eclipse microscope (Nikon, Melville, NY) for 3 random fields per sample and quantified using Image J software.

### 4.12 Immunofluorescence staining

Immunofluorescence staining was performed as described in Thadathil et al., (2021) with modifications. Briefly, liver cryosections (10 μm in thickness) were permeabilized, blocked and incubated with primary antibodies against P‐MLKL, albumin (Novus Biologicals), F4/80 (Novus Biologicals) over night at 4°C. This was followed by staining with the corresponding fluorescent tagged secondary antibodies for 1 h at room temperature: goat anti‐rabbit Alexa Fluor 647 (Thermo Fisher Scientific), donkey anti‐goat Alexa Fluor 488 (Thermo Fisher Scientific), goat anti‐rat Alexa Fluor 488 (Abcam). The nuclei were counter stained with DAPI (Thermo Fisher Scientific) and mounted with ProLong™ Diamond antifade mountant (Thermofisher scientific). All the imaging was acquired with a confocal microscope (Zeiss LSM 710) at 200× and 630× magnifications in five non‐overlapping fields per mouse.

Paraffin sections of liver were used for immunofluorescent staining for F4/80, CD68 and CD206. The primary antibodies used are F4/80 (Novus Biologicals), CD68 (Bio‐Rad) or CD206 (Abcam) and the secondary antibodies used are goat anti‐rat Alexa Fluor 488 (Abcam), donkey anti‐rabbit Alexa Fluor 488 (Abcam). Nuclei were counter stained with DAPI and the sections mounted with VectaMount AQ Aqueous Mounting Medium (Vector laboratories). Images were acquired with Nikon TE2000‐E fluorescent microscope. Fluorescent intensity (percentage area of green fluorescence) was calculated using Image J software (U.S. National Institutes of Health) from 3 random fields per sample.

### 4.13 Administration of Nec‐1s

Nec‐1s administration was performed as described previously (Mohammed et al., 2021). Daily consumption of Nec‐1s based on this protocol is reported to be 2.5–5 mg/day (Ito et al., 2016).

### 4.14 Serum alanine aminotransferase (ALT) measurement

Serum levels of ALT were measured using alanine transaminase colorimetric activity assay kit from Cayman Chemical Company (Ann Arbor, MI) following manufacturer’s instructions.

### 4.15 Statistical analyses

Ordinary one‐way ANOVA with Tukey's *post hoc* test and Student’s *t* test was used to analyze data as indicated. *P* < 0.05 is considered statistically significant.

## CONFLICT OF INTEREST

The authors declare no competing financial interests.

## AUTHOR CONTRIBUTIONS

S.M. performed the experiments, analyzed data, and prepared figures, N.T. performed immunofluorescence experiments and edited the manuscript, R.S. performed western blots for Nec‐1s study, E.H.N. performed all the real‐time qPCR, D.W. helped with animal studies, collaborated with B.F.M. for hydroxyproline assay, B.F.M. and A.R. gave critical comments and suggestion for the manuscript; and S.S.D designed the experiments, wrote and edited the manuscript.

## Supporting information

Figures S1‐S4

Table S1

Supplementary Material

## Data Availability

The data that supports the findings of this study are available in the manuscript and supplementary material of this article. Correspondence and requests for information should be addressed to S.S.D.
