# Supplementary figures and images for: Necroptosis contributes to chronic inflammation and fibrosis in aging liver
Source: Aging Cell. 2021 Nov 11;20(12):e13512. doi: 10.1111/acel.13512 (PMC8672775; doi:10.1111/acel.13512)

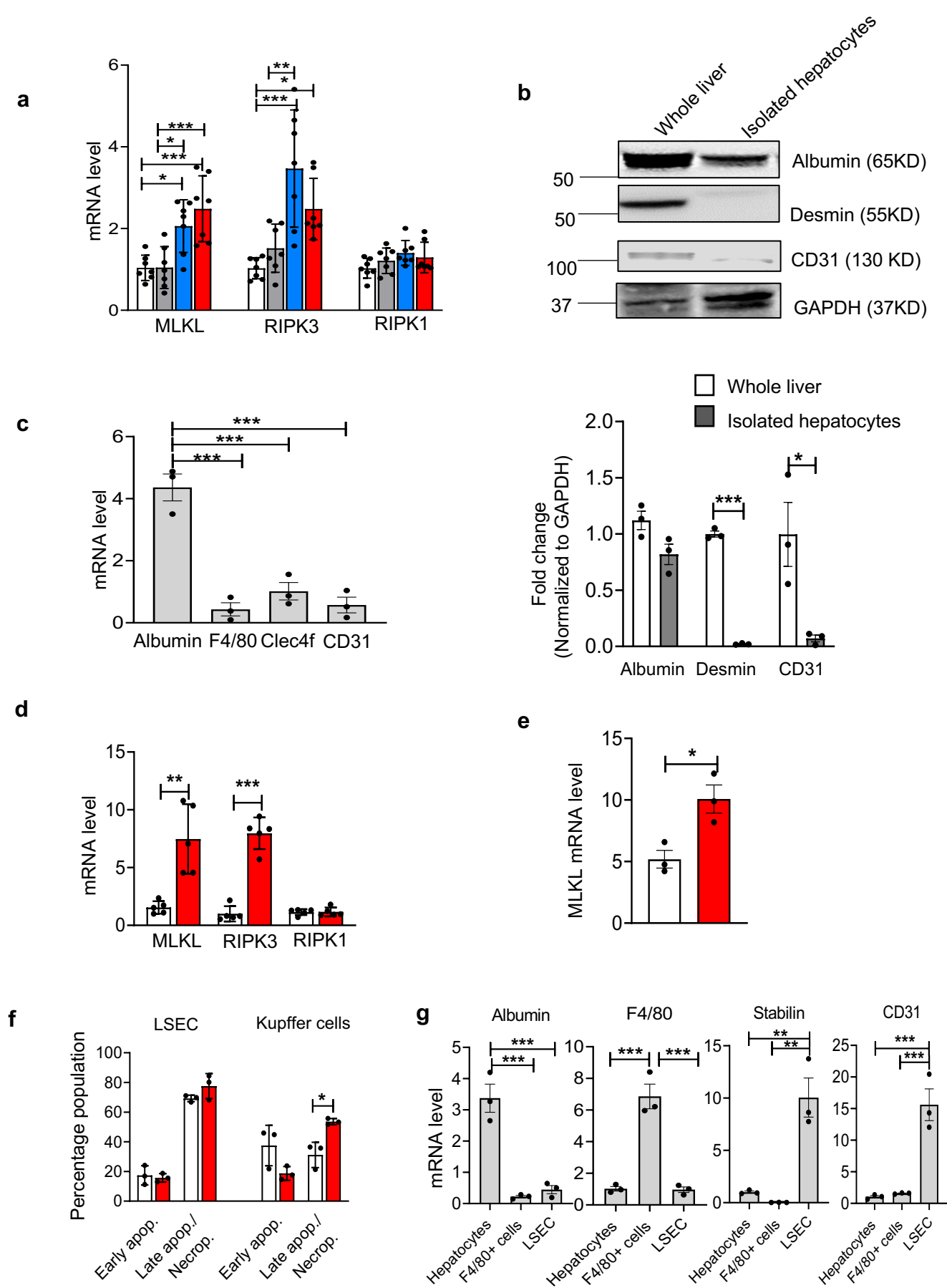

**Figure S1**

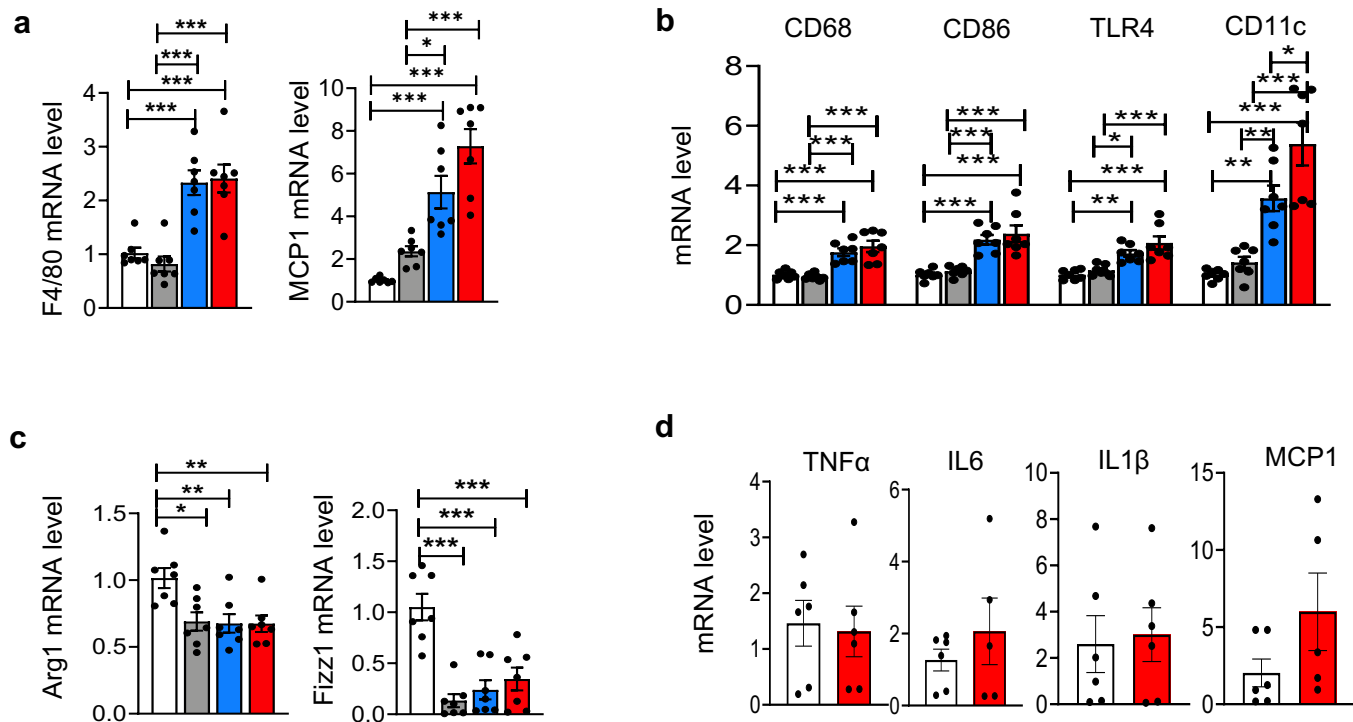

**Figure S2**

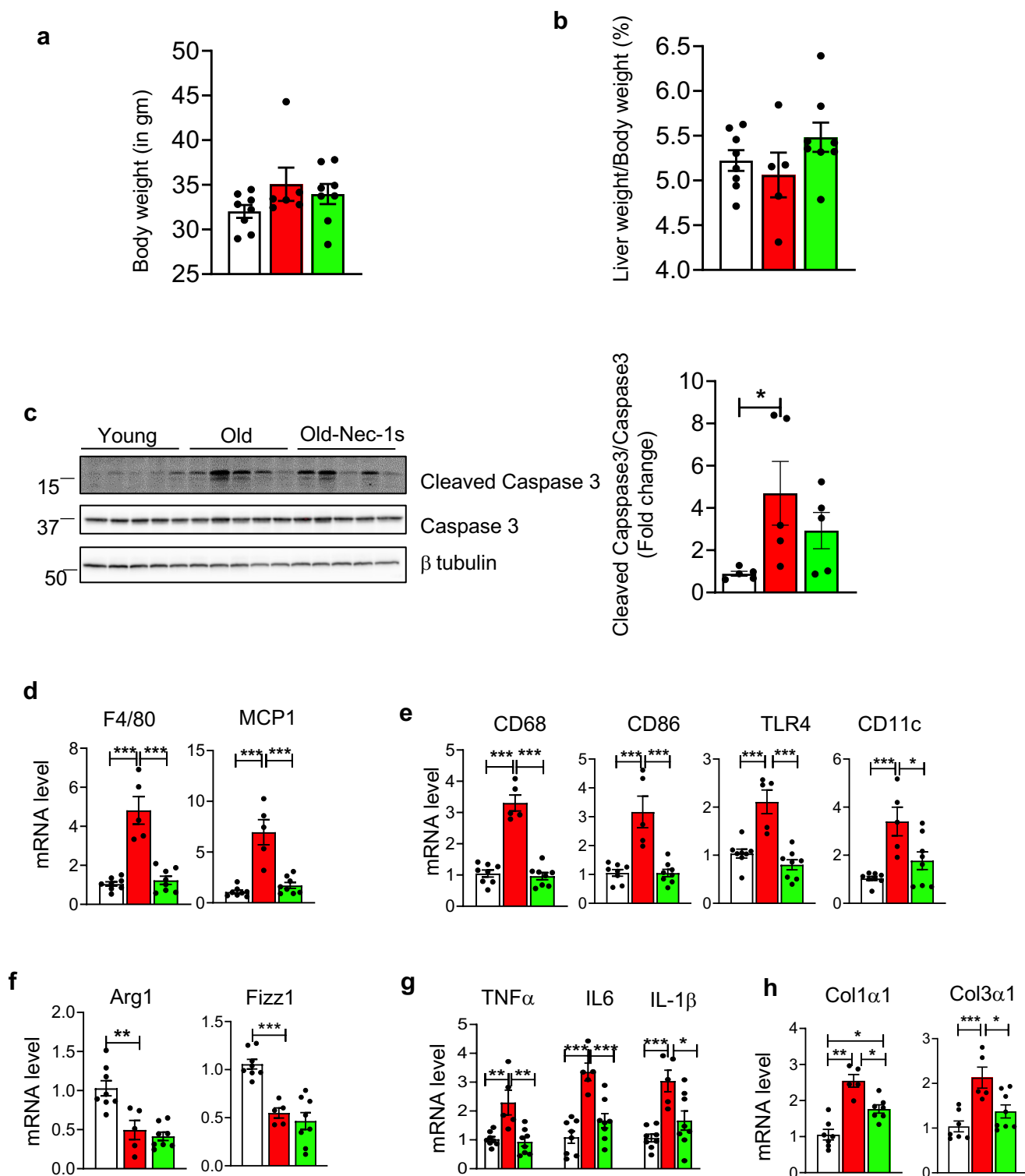

Figure S3

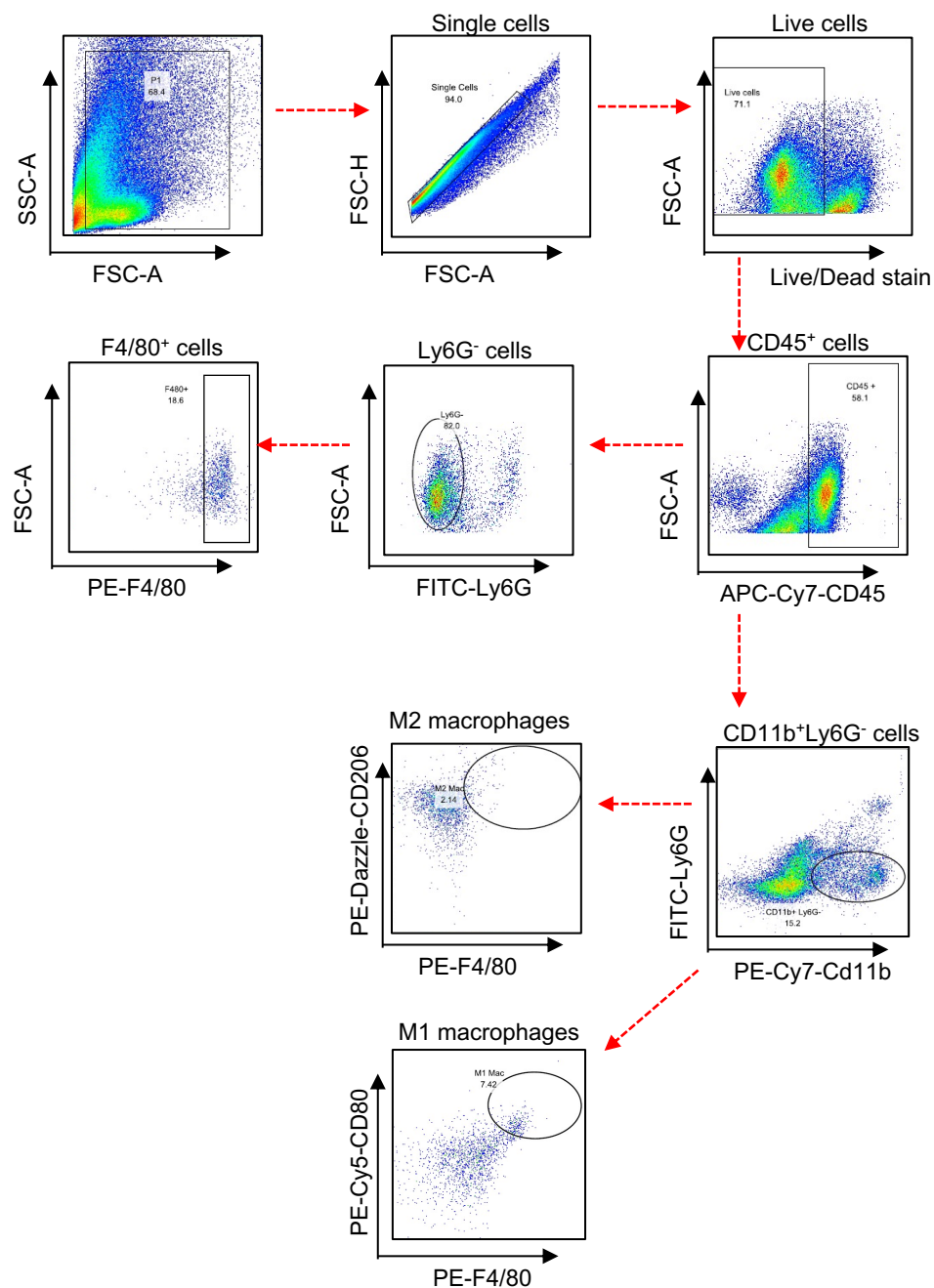

**Figure S4**

Supplement: Supplementary file 1 — Figures S1‐S4 [file ACEL-20-e13512-s002.pdf]
