# Supplementary material for: Necroptosis contributes to chronic inflammation and fibrosis in aging liver
Source: Aging Cell. 2021 Nov 11;20(12):e13512. doi: 10.1111/acel.13512 (PMC8672775; doi:10.1111/acel.13512)
Supplement: Supplementary file 2 — Table S1 [file ACEL-20-e13512-s003.pdf]

TABLE S1: LIST OF REAL TIME PCR PRIMERS

Gene	Forward sequence	Reverse sequence
Albumin	5'-GCGCAGATGACAGGGCGGAA-3'	5'-GTGCCGTAGCATGCGGGAGG-3'
Arg1	5'-CTCCAAGCCAAAGTCCTTAGAG-3'	5'-AGGAGCTGTCATTAGGGACATC-3'
CD11C	5'-CTGGATAGCCTTTCTTCTGCTG-3'	5'-GCACACTGTGTCCGAACTC-3'
CD31	5'-CTGGTGCTCTATGCAAGCCT-3'	5'-AGTTGCTGCCCATTCATCAC-3'
CD68	5'-CCACAGGCAGCACAGTGGAC-3'	5'-TCCACAGCAGAAGCTTTGGCCC-3'
CD86	5'-ACGATGGACCCCAGATGCACCA-3'	5'-GCGTCTCCACGGAAACAGCA-3'
Cdkn1a(p21^{Cip1})	5'-GTCAGGCTGGTCTGCCTCCG-3'	5'-CGGTCCCGTGGACAGTGAGCAG-3'
Cdkn2a(p16^{Ink4a})	5'-CCCAACGCCCCGAACT-3'	5'-GCAGAAGAGCTGCTACGTGAA-3'
Clec4f	5′-CTTCGGGGAAGCAACAACTC-3	5′-CAAGCAACTGCACCAGAGAAC-3′
Col1α1	5'-GCTCCTCTTAGGGGCCACT-3'	5'-CCACGTCTCACCATTGGGG-3'
Col3α1	5'-CTGTAACATGGAAACTGGGGAAA-3'	5'- CCATAGCTGAACTGAAAACCACC-3'
F4/80	5'-CCCCAGTGTCCTTACAGAGTG-3'	5'-GTGCCCAGAGTGGATGTCT-3'
Fizz1	5'-CCAATCCAGCTAACTATCCCTCC-3'	5'-CCAGTCAACGAGTAAGCACAG-3'
IL-6	5'-TGGTACTCCAGAAGACCAGAGG-3'	5'-AACGATGATGCACTTGCAGA-3'
Il-1β	5'-AGGTCAAAGGTTTGGAAGCA-3'	5'-TGAAGCAGCTATGGCAACTG-3'
MCP-1	5'-TTAAAAACCTGGATCGGAACCAA-3'	5'-GCATTAGCTTCAGATTTACGGGT-3'
MLKL	5′-CTGAGGGAACTGCTGGATAGAG-3′	5′-CGAGGAAACTGGAGCTGCTGAT-3′
RIPK3	5′-GAAGACACGGCACTCCTTGGTA-3′	5′-CTTGAGGCAGTAGTTCTTGGTGG-3′
RIPK1	5′-GACTGTGTACCCTTACCTCCGA-3′	5'-CACTGCGATCATTCTCGTCCTG-3'
Stabilin	5'-TGTCCAGACGGCTACATCAA-3'	5'-CCAGGGATATCCAGGACGTA-3'
TGFβ	5'-ACCATGCCAACTTCTGTCTGGGAC-3'	5'-ACAACTGCTCCACCTTGGGCTTG-3'
TLR4	5'-ATGGCATGGCTTACACCACC-3'	5'-GAGGCCAATTTTGTCTCCACA-3'
TNFα	5'-CACAGAAAGCATGATCCGCGACGT-3'	5'- CGGCAGAGAGGAGGTTGACTTTCT-3'
β- actin	5′-ATGGATGACGATATCGCTG-3′	5′-GTTGGTAACAATGCCATGTTC-3′
β-microglobulin	5′-CACTGACCGGCCTGTATGC-3′	5′-GGGTGGCGTGAGTATACTTGAAT-3′
HPRT	5'-CTGGTGAAAAGGACCTCTCG-3'	5'-TGAAGTACTCATTATAGTCAAGGGCA-3'
