# Supplementary material for: Necroptosis contributes to chronic inflammation and fibrosis in aging liver
Source: Aging Cell. 2021 Nov 11;20(12):e13512. doi: 10.1111/acel.13512 (PMC8672775; doi:10.1111/acel.13512)
Supplement: Supplementary file 3 — Supplementary Material [file ACEL-20-e13512-s001.pdf]

SUPPLEMENTARY FIGURES:

FIGURE S1 (a) Transcript levels of *Mlkl, Ripk3, Ripk1* in the livers of 7 (white bars), 12 (grey bars), 18 (blue bars), and 22 to 24-month-old mice (red bars) normalized to β-microglobulin and expressed as fold change. (b) Top: Immunoblots of whole liver extracts (white bars) and isolated hepatocytes (grey bars) for albumin, desmin, CD31 and GAPDH. Bottom: Graphical representation of the quantified blots normalized to GAPDH. (c) Transcript levels of albumin, F4/80, Clec4f and CD31 in the isolated hepatocyte fraction normalized to β-microglobulin and expressed as fold change. Data represented as mean $\pm$ SEM, * $p< 0.05$, ** $p< 0.005$, *** $p<0.0005$, n = 3/group. (d) Transcript levels of *Mlkl*, *Ripk3 and Ripk1* in hepatocytes from young (white bars) and old mice (red bars) normalized to β-microglobulin and expressed as fold change. (e) Transcript level of MLKL in the F4/80^{+} cells isolated from young (white bar) and old mice (red bar). (f) Graphical representation of the early apoptotic and late apoptotic/necroptotic population in the annexin/propidium iodide staining of LSEC and KC fraction obtained by MACS (g) Transcript levels of albumin (hepatocyte marker), F4/80 (macrophage marker), stabilin and CD31 (endothelial markers) in the cell fractions isolated by MACS. Data represented as mean $\pm$ SEM, * $p< 0.05$, ** $p< 0.005$, *** $p<0.0005$, n = 3/group.

FIGURE S2 (a) Transcript levels of F4/80 and MCP1 in the livers of 7 (white bars), 12 (grey bars), 18 (blue bars), and 22 to 24-month-old (red bars) mice normalized to β-microglobulin and expressed as fold change. Transcript levels of (b) CD68, CD86, TLR4 and CD11c (c) Arg1 and Fizz1 in the livers of 7 (white bars), 12 (grey bars), 18 (blue bars), and 22 to 24-month-old (red bars) mice normalized to β-microglobulin and expressed as fold change. (d) Transcript levels of TNFα, IL6, IL-1β and MCP-1 in LSEC isolated from young (white bars) and old (red bars). Data represented as mean $\pm$ SEM, * $p< 0.05$, ** $p< 0.005$, *** $p<0.0005$, n = 5-7/group.

FIGURE S3 Effect of Necrostatin-1s on body weight and liver weight. (a) The body weight and (b) percentage liver weight of young (7 months, white bars), old (24 months, red bars) and old mice treated with Nec-1s (24 months, green bars). (c) Left panel: Immunoblots of liver extracts prepared from young (7 months, white bars), old (24 months, red bars) and old mice treated with Nec-1s (24 months, green bars) for cleaved caspase-3, caspase 3 and β-tubulin. Right panel: Graphical representation of quantified blots normalized to β-tubulin. (d-h) Transcript levels of (d)

F4/80 and MCP1, (e) CD68, CD86, TLR4, and CD11C, and (f) Arg1, Fizz1 (g) TNFα, IL6, IL-1β (h) Col1α1, Col3α1 in the livers of young, old, and old-Nec-1s mice normalized to β-microglobulin and expressed as fold change. Data represented as mean ± SEM, * $p < 0.05$, ** $p < 0.005$, *** $p < 0.0005$, n = 5-7/group.

Figure S4 Gating strategy that was followed for the flow cytometry experiments.
